# Effect of comfortable nursing on postoperative nausea and vomiting in patients with idiopathic scoliosis after posterior orthopedic surgery

**DOI:** 10.3389/fsurg.2024.1395013

**Published:** 2024-07-03

**Authors:** Ziqi Chen, Chunyi Liu, Wenyue Chen

**Affiliations:** Division of Spine Surgery, Department of Orthopedic Surgery, Nanjing Drum Tower Hospital, Affiliated Hospital of Medical School, Nanjing University, Nanjing, Jiangsu, China

**Keywords:** comfort care, idiopathic scoliosis, nausea and vomiting, VAS, physical signs, depression, anxious

## Abstract

**Objective:**

To evaluate the effect of comfort nursing on postoperative nausea and vomiting in patients with idiopathic scoliosis undergoing posterior correction surgery.

**Methods:**

92 patients with idiopathic scoliosis were taken as the subjects and segmented into a control group and an experimental group (*n* = 46/each group). The former received routine care, while the latter one performed comfortable care. The observation period is 48 h after surgery. Record and compare the incidence, grade, frequency, and pain level of nausea and vomiting in both groups, as well as postoperative physical signs and symptoms, drug use, and postoperative recovery. Investigating the patient's satisfaction with nursing care. The research data is analyzed using SPSS26.0 software. *P *< 0.05 means statistical significance.

**Results:**

Within 48 h after surgery, the number of nausea and vomiting in the control is 24 and the experimental group is 8, with an incidence rate of 52% and 16%. The latter is significantly lower than that in the control. The average number of nausea and vomiting episodes in the control is 2.5, significantly higher than the 0.45 episodes in the experimental set. There is a significant difference in the frequency of nausea and vomiting/temperature and urine volume/scores of nausea, vomiting, dizziness, headache, decreased appetite, and discomfort between the two groups (*P *< 0.05).

**Conclusion:**

Comfortable care has a relieving effect on postoperative nausea and vomiting in patients with idiopathic scoliosis after posterior correction surgery. It can low down the incidence and frequency of nausea and vomiting, and reduce the score of related symptoms. Comfortable care can also help patients recover after surgery, increase dietary intake, and improve nutritional status. Comfortable care has a significant effect on postoperative nausea and vomiting in cases with idiopathic scoliosis undergoing posterior correction surgery, which can improve their postoperative recovery and quality of life.

## 1 Introduction

Idiopathic scoliosis is a common spinal disease. Its main feature is scoliosis and distortion of the spine, which seriously affects the patient's appearance and physical function (1). This disease usually begins to develop in children and adolescents, but it can also occur in adults (1). Scoliosis can cause asymmetry in the patient's body, such as uneven height of the shoulders and buttocks, resulting in significant appearance changes (1, 2). In addition to changes in appearance, idiopathic scoliosis may also cause a series of physical problems for patients. A curved spine may cause imbalance and tension in the back muscles, leading to back pain and stiffness. In severe cases, scoliosis of the spine may affect the function of internal organs, leading to breathing difficulties, heart compression, and other problems. The methods for treating idiopathic scoliosis vary depending on individual circumstances (2). Early diagnosis and treatment are crucial for children and adolescents to prevent the further development of scoliosis. In some severe cases, surgical correction of scoliosis may be necessary (2). To treat this disease, posterior orthopedic surgery has become one of the main treatment methods, correcting spinal deformities by implanting screws and rods (3). However, postoperative nausea and vomiting (P-NV) is one of the common complications in posterior orthopedic surgery, which badly affects the surgical recovery and quality of life (QOL) of patients (4, 5). Nausea and vomiting (N&V) not only cause physical discomfort to patients, but may also lead to complications such as electrolyte disorders, dehydration, and malnutrition (6, 7). In addition, N&V can also prolong the patient's hospitalized and recovery time, increase medical expenses and burden. To address this issue, comfort care, as a comprehensive nursing model, emphasizes the individualized needs of patients and comprehensive nursing measures (8). In the prevention and treatment of P-NV, comfortable care can reduce the occurrence of N&V by improving the patient's postoperative environment, providing appropriate sedative and analgesic measures, and adjusting the patient's diet and medication treatment reasonably. However, there is currently a lack of systematic research and analysis on the impact of comfort care on P-NV in patients with idiopathic scoliosis undergoing posterior correction surgery (PCS) (9, 10). Therefore, it is necessary to conduct more research to explore the impact of comfort care on P-NV in patients with idiopathic scoliosis undergoing PCS. This will help improve patients' surgical recovery and quality of life (QOL), decrease the complication occurrences, and reduce medical costs and burdens. Based on this, the purpose of paper is to explore the impact of comfort nursing on P-NV in patients with idiopathic scoliosis undergoing PCS, and to provide scientific basis and guidance for clinical practice. By analyzing the prevention and treatment effects of comfort nursing on P-NV, effective nursing strategies can be provided to improve patients' surgical recovery and QOL.

## 2 Methods

### 2.1 Research object

This study was conducted at the School of Medicine of Nanjing University, selecting 92 patients with scoliosis who underwent surgical treatment from January 2021 to December 2022 as the study subjects. The selection process must strictly comply with ethical standards (7). The inclusion criteria are as follows: ① The patient does not have contraindications to anesthesia; ② The patient has no history of drug allergy; ③ The patient voluntarily participates in the experiment and signs an informed consent form; ④ The patient's listening, speaking, reading, and writing abilities are normal. The exclusion criteria are as follows: ① The patient has other diseases that induce vomiting and nausea; ② The patient has a mental illness that affects communication; ③ The patient has other serious complications or illnesses. 92 subjects who met the criteria were randomly separated into two groups, the control group (C, *n* = 46) and the experimental group (E, *n* = 46).

### 2.2 Research method

All patients underwent spinal correction, bone graft fusion, and internal fixation, with the same surgical and anesthesia methods. Among them, the C's patients received routine care, including education on anesthesia and surgical related knowledge, adverse reactions and corresponding treatment methods of postoperative analgesics, treatment methods for P-NV, and dietary education. Dietary education includes fasting for 6 h before surgery, fasting for 4 h, and eating for 6 h after surgery, with food gradually transitioning from liquid to regular (11).

The E's patients received comfort nursing interventions. ① Preoperative individualized psychological care: comprehensively evaluate the condition and needs of patients and their families, and provide targeted psychological counseling. Use psychological relaxation techniques to help patients relieve tension and alleviate possible N&V symptoms after surgery. Briefly explain surgical related knowledge to family members, remind them to maintain a good emotional state, and provide emotional support and comfort to patients. ② Dietary care: Guide patients to consume a light and easily digestible diet before surgery. Before surgery, stop eating solid foods and drink carbohydrate drinks or energy mixtures. Try drinking a small amount of warm water after surgery. If there are no symptoms of nausea or vomiting, enter a light diet after 4 h, and then give a semi liquid diet that is easy to digest. Avoid dairy products, soy products, and desserts, and avoid fried foods. Provide a balanced and delicious diet based on the patient's taste and health needs. ③ Pay close attention to patients: observe changes in blood pressure, follow medical advice, and ensure sufficient capacity. Inhale oxygen according to medical advice (with a conventional oxygen concentration of 3 L/min). ④ Positioning: Raising the head of the bed by 10°–30° after the patient's vital signs have stabilized can alleviate pain and reduce the occurrence of N&V. After 4 h of surgery, roll over on the axis, and the best lateral position is 30°–45° oblique position. When changing positions, move slowly and gently. ⑤ Acupoint care: Teach patients to press the Neihegu and Neiguan acupoints with their fingers, and press for 3 min in the morning and evening respectively, preferably with a slight sensation of soreness and swelling in the local area. Abdominal massage and warm compress on the abdomen can promote intestinal peristalsis and increase comfort. ⑥ Keep the environment comfortable: To maintain the comfort of the environment, the indoor temperature should be appropriate and there should be a good ventilation system to ensure air circulation. In addition, the levels of noise and light should also be within an acceptable range to avoid unnecessary interference with patients. To provide a comfortable bed, comfortable mattresses, pillows, and sheets should be chosen, and the bed should be kept clean and tidy. In addition, appropriate lighting should be provided based on the individual needs and types of activities of the patient to avoid glare or dim lighting causing discomfort to the patient's vision. Finally, to ensure patient comfort, soft, breathable, and fitting clothing should be provided, and the patient's needs should be met as much as possible. ⑦ Providing emotional support: Based on establishing good communication and trust relationships with patients, medical staff provide emotional support and comfort to patients through listening, understanding, and caring, helping them reduce their psychological burden, enhance their coping ability, and promote rehabilitation and mental health. ⑧ Provide activities and entertainment: Provide patients with appropriate activities and entertainment, such as reading, listening to music, watching movies, etc., to reduce stress and improve mood (12, 13).

### 2.3 Observed indicators

General information: age, gender, body mass index (BIM), length of hospital stay, and duration of treatment. The N&V incidence in patients was evaluated according to World Health Organization standards: Level I: no N&V; Level II: Mild-nausea, abdominal discomfort, but no-vomiting; Level III: Obvious N&V, but no gastric contents vomited; Level IV: Severe vomiting, vomiting of gastric juice and other contents, and hard to control without medication. The frequency of N&V was obtained based on nursing records, and the pain degree was evaluated by visual analogue scale (VAS). The higher the score, the stronger the pain (14). The patient's postoperative physical characteristics include body temperature, blood pressure, heart rate, urine volume, and respiratory rate. The related symptoms of P-NV in patients include nausea, vomiting, dizziness, headache, decreased appetite, bloating, and discomfort. The use of medication for P-NV in patients with idiopathic scoliosis undergoing PCS should be determined based on the patient's specific situation and doctor's advice, including anti-nausea drugs, sedatives, analgesics, and oral or intravenous fluids. The postoperative recovery and recovery of gastrointestinal function in patients can be evaluated by recovery time, dietary intake, nutritional status, and gastrointestinal function indicators (15). The satisfaction level of patients with nursing can be divided into three types: very satisfied, satisfied, average, and dissatisfied. Very satisfied and satisfied together constitute patient satisfaction. The QOL after discharge includes seven aspects: physiological activity, emotional awareness, physical discomfort, mental health, sleep quality, anxiety or depression, and overall health. The study uses a QOL rating scale to quantitatively compare the QOL after discharge in two groups. The higher the score, the greater the QOL after discharge.

### 2.4 Statistical methods

The research data was analyzed using SPSS26.0 software (SPSS, Inc., Chicago, IL, USA). The counting data is described using various types of quantities and tested using chi square distribution. The measurement data is described using mean plus minus standard deviation, which conforms to a normal distribution and adopts *t*-test. Using rank sum test to test the median and upper and lower quartile representations. If *P *< 0.05, it indicates statistical significance (16).

## 3 Results

### 3.1 General information of two groups of patients

This study randomly divided 92 study subjects who met the standards into 2 groups, the C and E, with 46 in each group. As shown in Table 1, the general information of patients includes age, gender, BIM, length of hospital stay, and surgical time. The male/female ratios in the C and the E were 14/32 and 18/28, respectively, with an average age of 15.60 ± 3.67 years/16.52 ± 3.60 years. The BMI is 17.81 ± 1.73 kg/m^2^ and 18.14 ± 1.53 kg/m^2^, respectively. The anesthesia method is general anesthesia. The surgical time was 3.11 ± 0.78 h and 3.23 ± 0.97 h, respectively. The hospitalization time was 8.34 ± 1.05 days and 8.50 ± 1.16 days, respectively. The difference was meaningless in gender, age, BMI, surgical time, and hospital stay among patients, and there was *P *> 0.05 between the groups, indicating comparability between the two groups.

**Table 1:** General information.

Baseline information	Group C (*n* = 46)	Group E (*n* = 46)	Statistical value	*P*
Gender (Male/Female)	14/32	18/28	*χ*^2^ = 0.040	0.981
Age (years)	15.60 ± 3.67	16.52 ± 3.60	*t *= 3.642	0.161
BMI (kg/m^2^)	17.81 ± 1.73	18.14 ± 1.53	*t *= 2.128	0.345
Type of anesthesia	General anesthesia	General anesthesia	/	/
Operative time (h)	3.11 ± 0.78	3.23 ± 0.97	*t *= 2.126	0.435
Hospital stay (days)	8.34 ± 1.05	8.50 ± 1.16	*t *= 3.125	0.191

### 3.2 Incidence of N&V in patients

**Figure 1:**
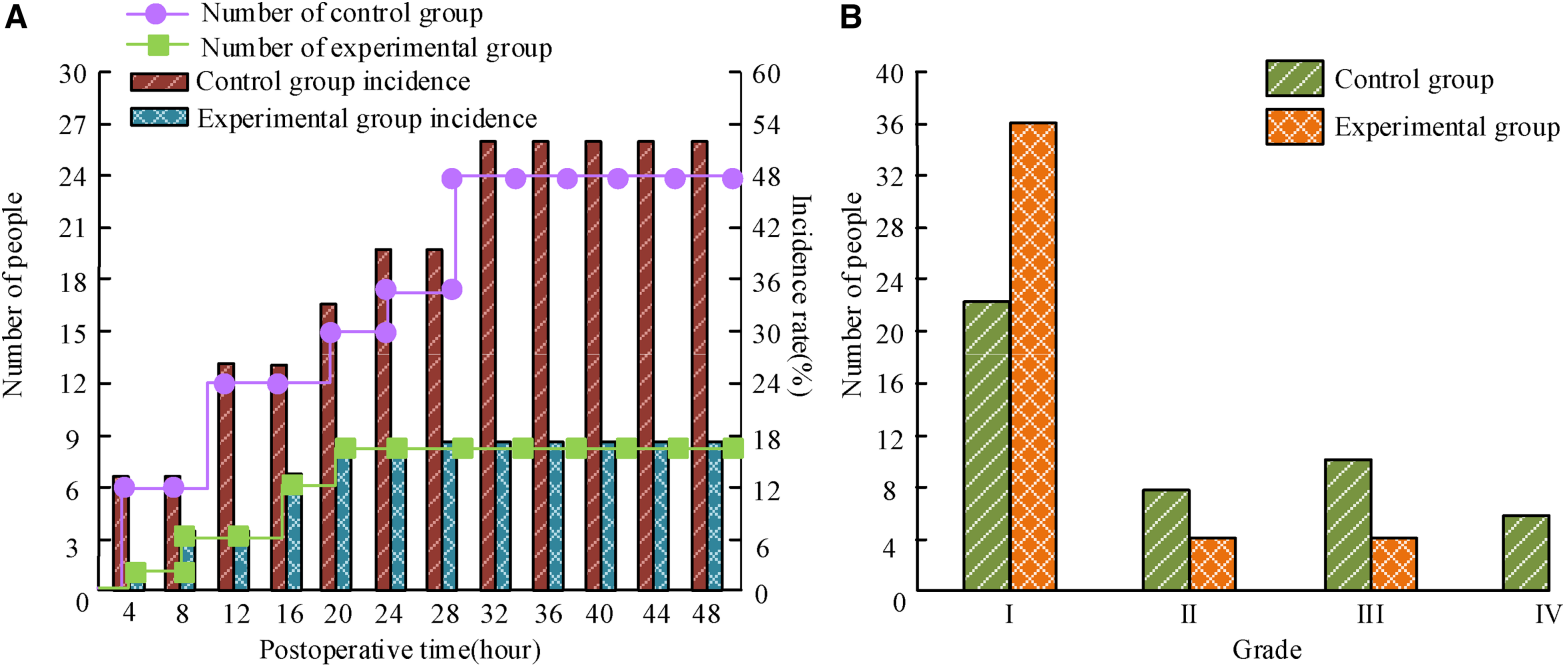
The average age and course of illness of the three groups of children. (**A**) Incidence of nausea and vomiting; (**B**) degree of nausea and vomiting.

The incidence and level of N&V in patients are shown in Figure 1. Within 48 h after surgery, the number of N&V in C was 24, with an incidence rate of 52%. The number of people with grade II, III, and IV N&V was 8, 10, and 6, respectively. The number of people in the experimental group experiencing N&V was 8, with an incidence rate of 16%. The number of people with grade II, III, and IV N&V was 4, 4, and 0, respectively, significantly lower than the C (*P *< 0.05).

### 3.3 Number of P-NV and degree of pain in two groups of patients

**Figure 2:**
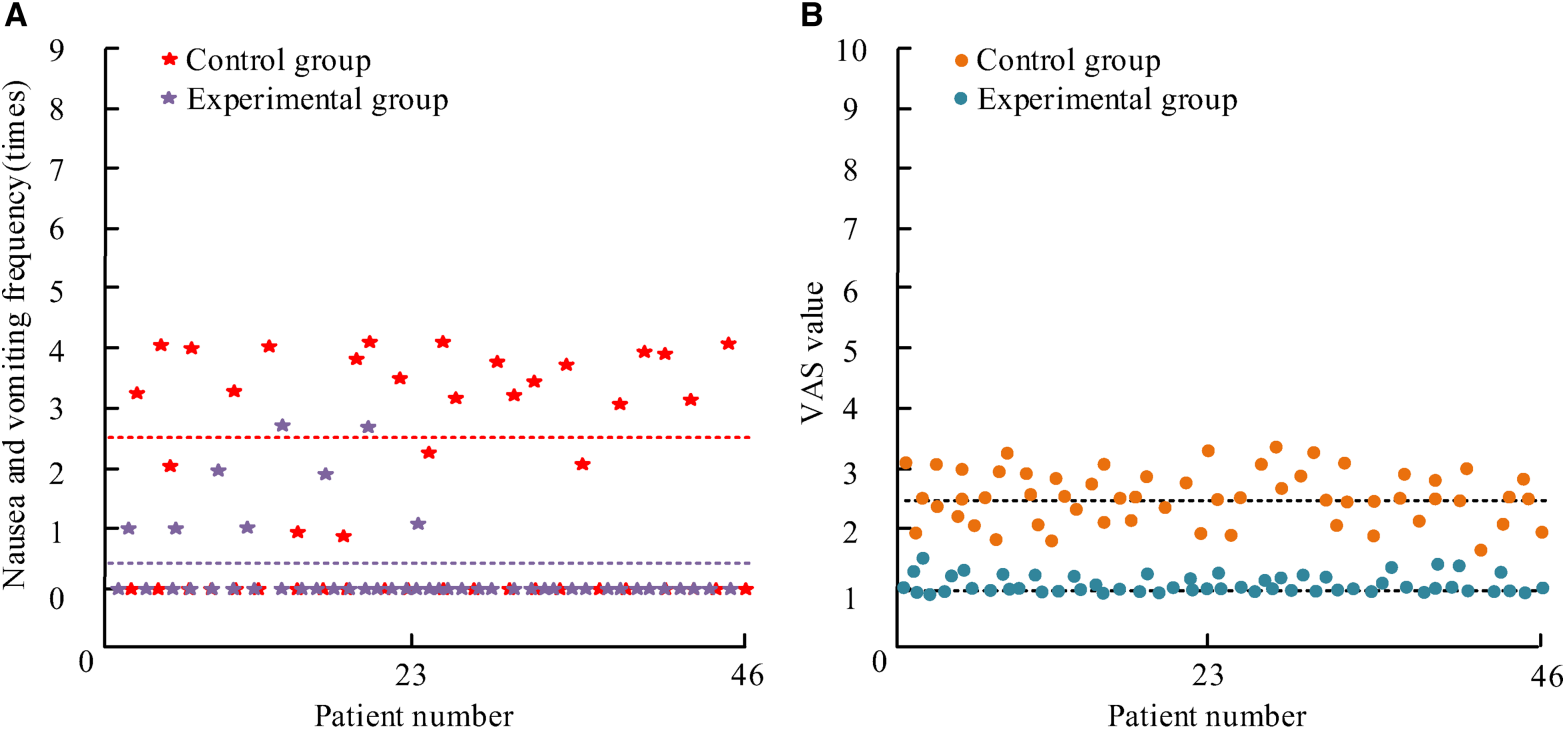
The average age and course of illness of the three groups of children. (**A**) Nausea and vomiting frequency; (**B**) pain level of nausea and vomiting.

The N&V frequency in the patient was determined based on nursing records, and the degree of pain was evaluated by VAS, as shown in Figure 2.

Figure 2A shows the frequency of N&V: the average frequency of N&V in C was 2.5 times, significantly higher than the 0.45 times in the experimental group. There was *P *< 0.05. Figure 2B shows the postoperative VAS score. The distribution of VAS scores in the C is within the (1,4) interval, while the E is within the (0,2) interval. Table 2 is the comparative data of VAS scores among patients [M (P25, P75)].

**Table 2:** Comparison of VAS scores between two groups [M (P25, P75)].

Group	Before treatment	After treatment	*Z*	*P*
Control group	4 (3,5)	2.4 (1.75,3.56)	−4.912	0.000
Experimental group	4 (3,5)	1 (0,2)	−4.762	0.000
*Z*	−0.769	−2.357	/	/
*P*	0.447	0.032	/	/

In Table 2, *P *> 0.05 before treatment indicates comparability. After treatment, the VAS scores within and between the two groups are *P *< 0.05. Both groups of patients show a decrease in VAS scores, indicating that both nursing plans can alleviate P-NV pain. The decrease in the E is 1 (0, 2), while the decrease in the control group is 2.4 (1.75, 3.56). The decrease in the experimental group is significant.

### 3.4 Postoperative physical signs of two groups of patients

As shown in Table 3, the postoperative physical characteristics of the patient include body temperature, blood pressure, urine volume, heart rate, and respiratory rate. The body temperatures of the C and E are 36.8 ± 0.8 °C and 36.1 ± 0.2 °C, respectively. The systolic blood pressure is 103.6 ± 18.2 mmHg and 98.4 ± 9.3 mmHg, respectively. The diastolic blood pressure is 86.1 ± 6.3 mmHg and 73.6 ± 5.6 mmHg, respectively. The heart rates were 83 ± 3.6 bpm and 70 ± 4.5 bpm, respectively. The urine volume is 27.3 ± 9.6 ml/h and 28.3 ± 10.2 ml/h, respectively. The respiratory rates are 15 ± 3.6 bpm and 16 ± 4.2 bpm. There are significant differences in these several indicators with *P *> 0.05, while there are significant differences in body temperature and urine volume between the groups (*P *< 0.05).

**Table 3:** Comparison of postoperative physical characteristics.

Physical signs	Control group (*n* = 46)	Experimental group (*n* = 46)	Statistical value	*P*
Temperature (°C)	36.8 ± 0.8	36.1 ± 0.2	*t *= 4.365	0.043
Blood pressure (mmHg)	103.6 ± 18.2;86.1 ± 6.3	98.4 ± 9.3;73.6 ± 5.6	*t *= 1.254	0.235
Heart rate (bpm)	83 ± 3.6	70 ± 4.5	*t *= 1.458	0.354
Urine output (ml/h)	27.3 ± 9.6	28.3 ± 10.2	*t *= 4.636	0.045
Respiratory rate (bpm)	15 ± 3.6	16 ± 4.2	*t *= 3.214	0.156

### 3.5 Symptoms related to P-NV

**Figure 3:**
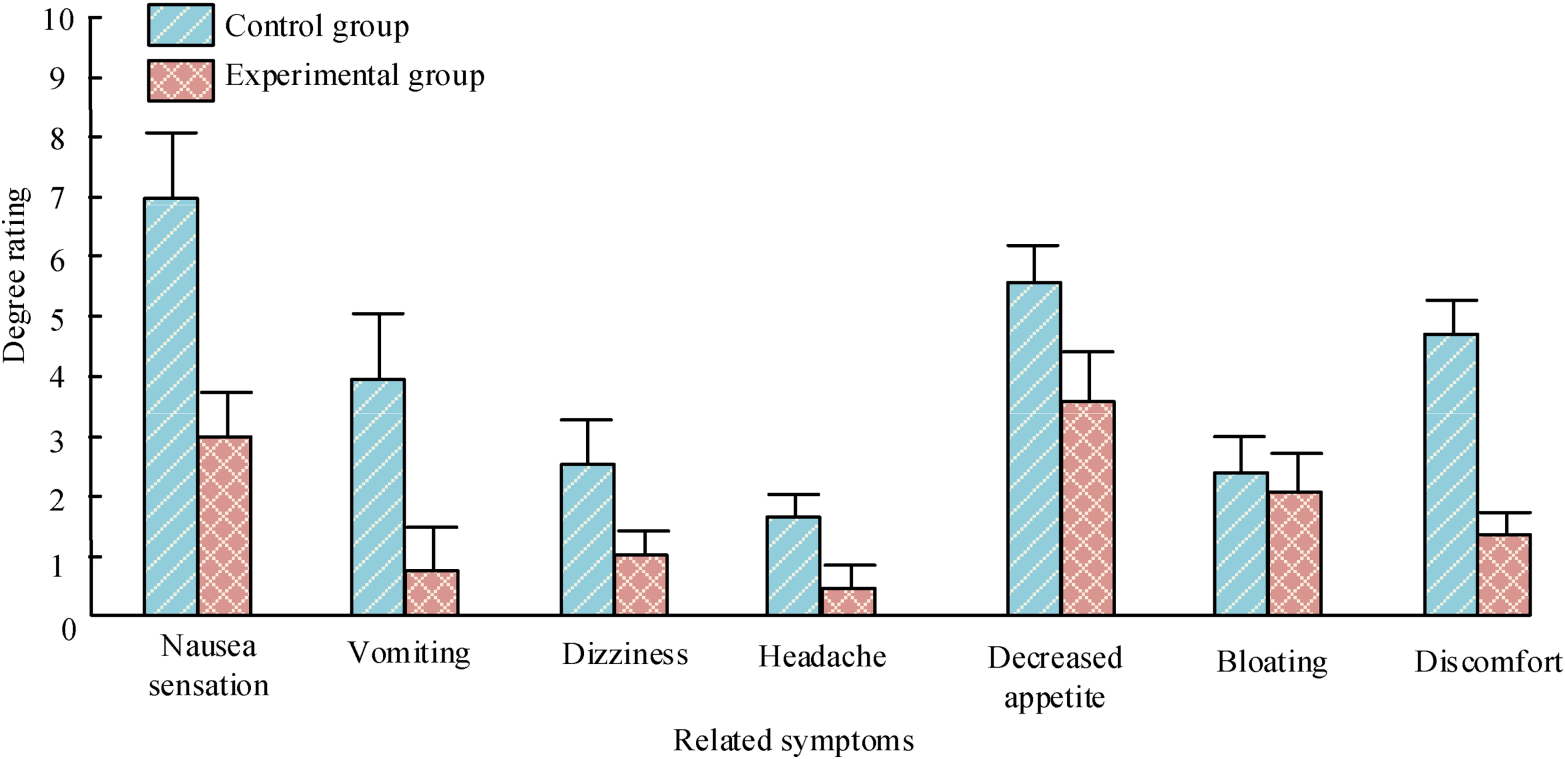
Comparison of N&V related symptoms between two groups of patients.

As shown in Figure 3, the related symptoms of P-NV in patients include nausea, vomiting, dizziness, headache, decreased appetite, abdominal distension, and discomfort. The distinction in abdominal distension scores among patients is not significant, with *P *> 0.05. There is a significant difference in the scores of various symptoms between the two groups of patients (*P *< 0.05).

### 3.6 P-NV medication use in two groups

**Figure 4:**
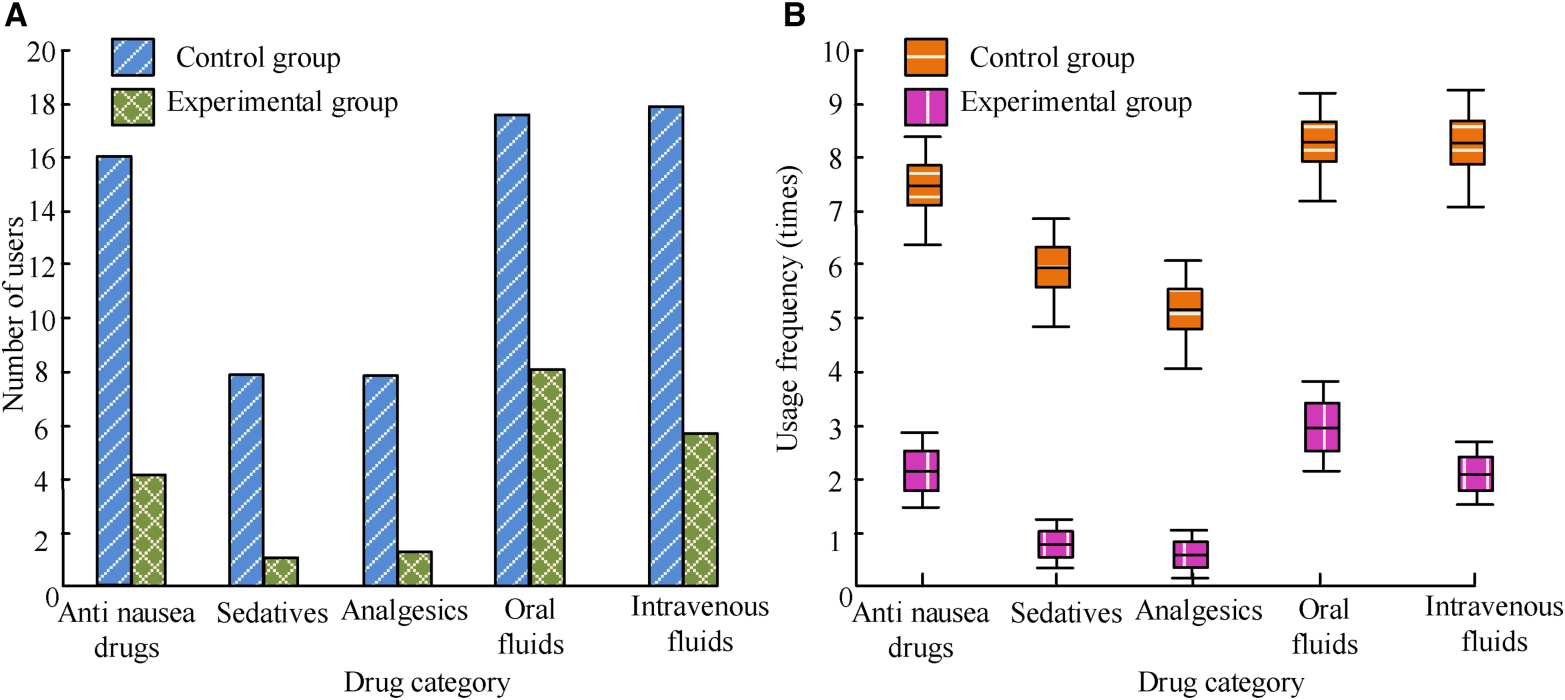
P-NV medication use in patients. (**A**) Number of people; (**B**) usage frequency.

As shown in Figure 4, the usage of medication for P-NV in patients with idiopathic scoliosis undergoing PCS should be determined based on the patient's specific situation and doctor's advice, including anti nausea drugs, sedatives, analgesics, and oral or intravenous fluids. The number and frequency of drug use in E are greatly lower than those in C, and the differences between the groups are *P *< 0.05.

### 3.7 Postoperative recovery and rehabilitation of gastrointestinal function in two groups of patients

The postoperative recovery and recovery of gastrointestinal function in patients can be evaluated by recovery time, dietary intake, nutritional status, and gastrointestinal function indicators. In Table 4, the comparison of recovery time, dietary intake, and nutritional status of patients shows *P *< 0.05. The gastrointestinal function and rehabilitation of the E are significantly better than C. Note: In the statistics of blood vitamins and blood electrolytes, R/T represents the proportion of normal test items to the total number of test items.

**Table 4:** Postoperative recovery and rehabilitation of gastrointestinal function.

Physical signs	Control group (*n* = 46)	Experimental group (*n* = 46)	Statistical value	*P*
Recovery time (days)	13.6 ± 6.3	9.6 ± 4.6	*t *= 2.456	0.031
Dietary intake (kcal/day)	1,500 ± 36.5	1,900 ± 40.3	*t *= 3.178	0.041
Patient weight gain (kg)	1.25 ± 0.85	2.01 ± 0.98	*t *= 2.178	0.024
Blood protein (g/L)	59.3 ± 6.5	65 ± 7.3	*t *= 3.125	0.036
Blood vitamins (R/T)	7.3/9	8.6/9	*χ*^2^ = 0.245	0.046
Blood electrolytes (R/T)	6.2/7	6.9/7	*χ*^2^ = 0.136	0.044

### 3.8 Satisfaction of two groups of patients with care

**Figure 5:**
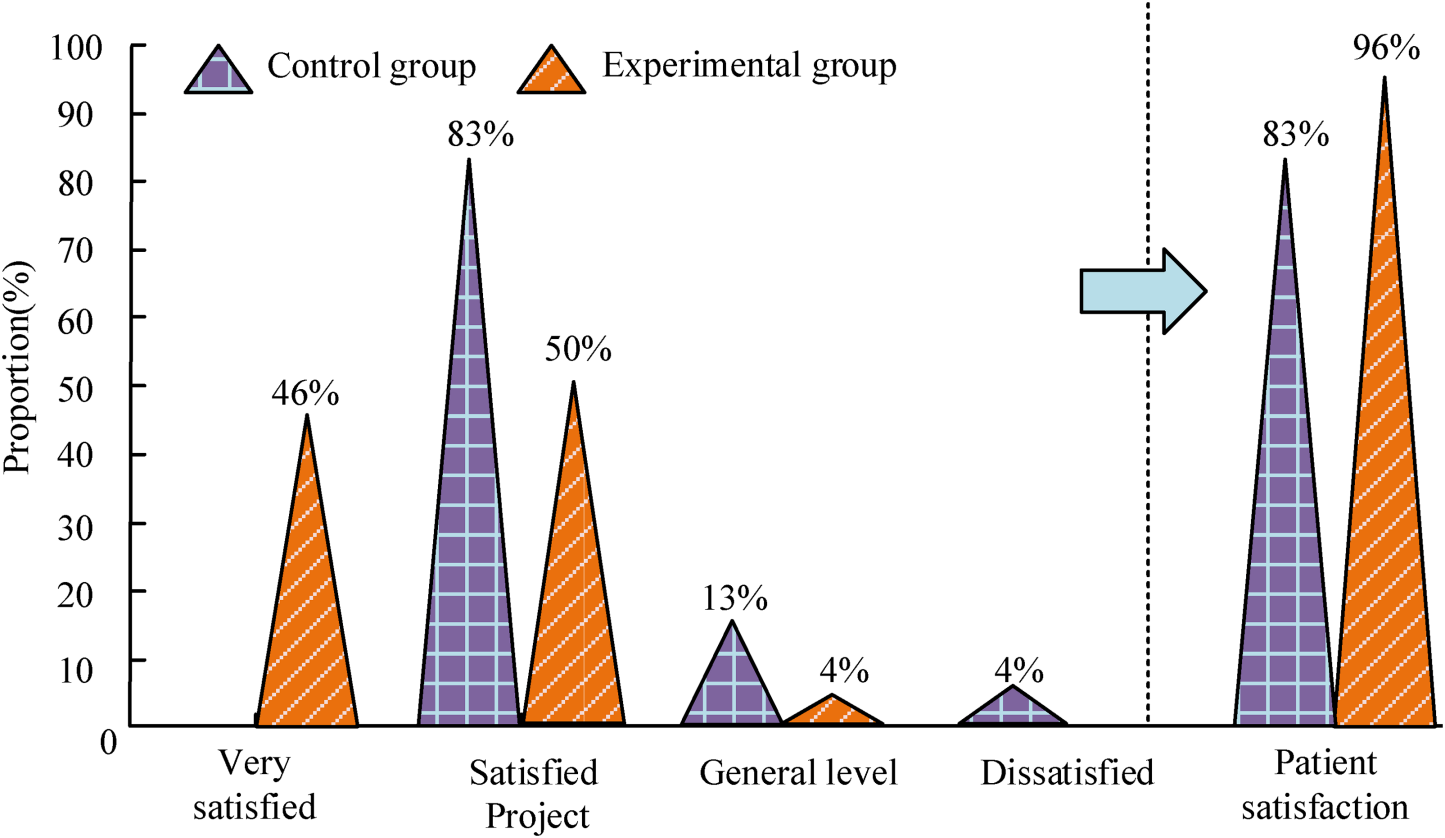
Satisfaction of two groups of patients with care.

The satisfaction level of patients with nursing can be divided into three types: very satisfied, satisfied, average, and dissatisfied. Very satisfied and satisfied together constitute patient satisfaction. The satisfaction with nursing is shown in Figure 5. Among the control group of patients, 38 are satisfied, with 6 being generally satisfied and 2 being dissatisfied. The patient satisfaction rate is 83%. Among the experimental group of patients, 21 are very satisfied, 23 are satisfied, accounting for 2 in general, and 0 are dissatisfied. Patient satisfaction is 96%. It can be seen that the patients' satisfaction in E is obviously higher than that of C.

### 3.9 Comparison of QOL between two groups after discharge

The QOL situation includes seven aspects: physiological activity, emotional awareness, physical discomfort, mental health, sleep quality, anxiety or depression, and overall health. As shown in Table 5, this study uses the QOL Rating Scale to quantitatively compare the QOL after discharge of patients. Higher scores represent better QOL after discharge. The physiological activity, emotional awareness, physical discomfort, mental health, sleep quality, anxiety or depression, and overall health scores of the control group are 70.6 ± 6.2, 75.4 ± 5.6, 76.3 ± 6.3, 66.6 ± 5.2, 66.5 ± 5.6, 69.3 ± 3.6, and 70.4 ± 5.4, respectively. The scores of the experimental group are 78.8 ± 5.4, 86.9 ± 4.9, 87.1 ± 6.9, 77.9 ± 5.6, 80.1 ± 3.5, 81.2 ± 6.4, and 84.6 ± 6.2, respectively. The QOL scores of E after discharge are significantly exceeded than C. The differences in various scores are *P *< 0.05.

**Table 5:** Comparison of QOL of patients after discharge.

QOL situation	Control group (*n* = 46)	Experimental group (*n* = 46)	*t*	*P*
Physiological activities	70.6 ± 6.2	78.8 ± 5.4	6.154	0.016
Emotional awareness	75.4 ± 5.6	86.9 ± 4.9	9.366	0.036
Physical discomfort	76.3 ± 6.3	87.1 ± 6.9	7.265	0.024
Mental health	66.6 ± 5.2	77.9 ± 5.6	9.014	0.039
Sleep quality	66.5 ± 5.6	80.1 ± 3.5	9.127	0.015
Anxiety or depression	69.3 ± 3.6	81.2 ± 6.4	8.035	0.003
Overall health	70.4 ± 5.4	84.6 ± 6.2	8.127	0.004

## 4 Discussion

In this study, our findings showed that the occurrence of P-NV in idiopathic scoliosis is related to multiple factors such as the surgery itself, anesthetic drugs, postoperative analgesics, individual differences in patients, and psychological factors. Through comprehensive comfort nursing measures, the occurrence of P-NV can be prevented and reduced, and the comfort and rehabilitation effect of patients can be improved.

Idiopathic scoliosis is a type of scoliosis that occurs during growth and development with unclear causes, with females accounting for 60%–80% (17). After surgery for idiopathic scoliosis, patients may experience symptoms of N&V. This may lead to inhalation pneumonia, dehydration, electrolyte disorders, and wound bleeding, thereby affecting the patient's recovery and prolonging hospital stay, increasing medical costs (18). At present, the causes of P-NV symptoms are not very clear, but research has shown that factors such as age, gender, surgery, anesthesia, medication, and psychology jointly lead to the N&V occurrence symptoms in patients. Some studies have also found that communicating with patients about anesthesia related complications before surgery can alleviate their preoperative anxiety to some extent, but excessive explanations may have psychological implications for patients, thereby strengthening their psychological response to N&V (19). Therefore, when conducting education, efforts should be made to downplay the content of P-NV education and reduce patients' fear. At the same time, guiding patients to shorten the preoperative fasting time and providing them with water as early as possible after surgery can not only reduce discomfort such as thirst and hunger, but also increase intestinal peristalsis, promote nutrient absorption and recovery. Clinical studies have shown that shortening fasting time and early water intake can significantly improve discomfort such as thirst, reduce the incidence of postoperative stress reactions, improve patients' subjective feelings, and accelerate recovery. In addition, studies have shown that hypotension may lead to transient hypoxia and gastrointestinal mucosal hypoperfusion during vomiting (20). Therefore, maintaining normal blood pressure levels and oxygen inhalation can improve local hypoxia, maintain normal intestinal perfusion, maintain mucosal acid-base balance, and thus reduce the occurrence of N&V. Therefore, it is crucial to monitor and maintain blood pressure after surgery, while ensuring that patients receive normal oxygen intake. Comfortable care plays a crucial role in the prevention and management of P-NV in idiopathic scoliosis. Comfortable care includes comprehensive nursing measures such as diet and water management, pain control, postoperative sedation, and psychological support for patients. Reasonable dietary management and water supplementation can reduce gastrointestinal discomfort and improve patient comfort. Adequate pain control can alleviate the patient's sense of pain, thereby reducing the occurrence of N&V. Postoperative sedation and psychological support can alleviate patients' anxiety and fear, and reduce the risk of N&V (21).

According to the research results, the incidence of P-NV in E was significantly lower than that in C, and the frequency of N&V and the degree of pain in E were also lower than those in C. In addition, there was a significant difference in postoperative body temperature and urine volume between the two groups of patients (*P *< 0.05), while there was no distinction in blood pressure, respiratory rate and heart rate (*P *> 0.05). In terms of P-NV related symptoms, there was no significant difference in abdominal distension scores in both groups, but there was a difference in nausea, vomiting, dizziness, headache, loss of appetite, and discomfort scores (*P *< 0.05). Meanwhile, the drug use, physical recovery, nursing satisfaction, and QOL after discharge in the E were significantly better than those in C. This study shows that patients with idiopathic scoliosis who receive comfortable care have shown significant improvements in P-NV. The incidence of N&V is significantly reduced, and the N&V degree in patients is also relatively mild. This indicates that comfort care has potential clinical significance in reducing P-NV in patients with idiopathic scoliosis after PCS, and these results are consistent with previous studies (22). The mechanism by which comfortable care may affect P-NV in patients with idiopathic scoliosis undergoing PCS is not yet clear. However, some studies have provided some possible explanations (23).

## 5 Conclusion

In summary, comfortable care can reduce the inflammatory response caused by surgical trauma, thereby reducing the P-NV incidence. In addition, comfortable care can also improve postoperative pain management for patients, reduce the use of analgesics, and further reduce the risk of N&V. Although the outcomes of this manuscript and existing research support the positive impact of comfort care on P-NV in patients with idiopathic scoliosis undergoing PCS, there are also some limitations in this paper. First, the sample size is relatively small and there may be selection bias. In addition, without further analysis of the specific components of comfort care, it is impossible to determine which factors play a major role in improving N&V. Future research can further explore the specific mechanisms of comfort care and its best practices in managing P-NV in patients with idiopathic scoliosis undergoing PCS. In addition, larger scale and randomized controlled clinical trials can be conducted to further validate the research results and provide stronger evidence support.

## Data Availability

The original contributions presented in the study are included in the article/Supplementary Material, further inquiries can be directed to the corresponding author.
